# Codevelopment of a Digital Screening and Intervention Tool to Improve Lifestyle Habits in Children: Focus Group Study With Parents and Clinicians

**DOI:** 10.2196/84304

**Published:** 2026-06-26

**Authors:** Alice Moyne, Tamara Perez, Trevor van Mierlo, Maryam Kebbe, Olivier Drouin

**Affiliations:** 1 Department of Nutrition Université de Montréal Montréal, QC Canada; 2 Centre de recherche Azrieli du Centre Hospitalier Universitaire Sainte-Justine Centre Hospitalier Universitaire Sainte-Justine Montréal, QC Canada; 3 Département Prévention Cancer Environnement Centre Léon Bérard Lyon, Auvergne-Rhône-Alpes France; 4 Evolution Health Toronto, ON Canada; 5 Faculty of Kinesiology University of New Brunswick Fredericton, NB Canada; 6 Department of Pediatrics Université de Montréal Montréal, QC Canada; 7 Department of Pediatrics Division of Pediatrics Centre Hospitalier Universitaire Sainte-Justine Montréal, QC Canada; 8 Department of Social and Preventive Medicine School of Public Health Université de Montréal Montréal, QC Canada

**Keywords:** pediatrics, screening, intervention, lifestyle behavior, qualitative study, digital tool

## Abstract

**Background:**

Chronic preventable diseases represent a major burden in Canada, often rooted in unhealthy behaviors established during childhood. Despite recommendations for routine screening, most children are not assessed due to clinical barriers. This paper presents the early development of Project DISCO, a self-administered, digital preappointment tool to screen for and support healthy lifestyle behaviors, including physical activity, sleep, nutrition, and screen time, in children aged 2 to 12 years.

**Objective:**

This study aimed to explore clinicians’ and parents’ needs and past experiences with health technology; integrate their insights into the development of a digital tool for promoting health behaviors; and evaluate the usability, relevance, and acceptability of this tool.

**Methods:**

Three semistructured focus group and interview sessions were conducted with primary care clinicians and parents. Participants were recruited at the Groupe de Médecine de Famille Universitaire Saint-Hubert using purposive and convenience sampling: clinicians were recruited internally, whereas parents of children aged 2 to 12 years were invited via emails sent by clinic staff. Data were analyzed using a combination of inductive and deductive approaches. Findings informed iterative refinements throughout the codevelopment of the digital tool for promoting healthy lifestyle behaviors in children.

**Results:**

A total of 8 participants took part in 6 discussions, including 5 parents of children aged 3 to 12 years and 3 primary care clinicians. Hybrid thematic analysis identified six themes: (1) the potential of a digital tool to promote healthy habits in pediatrics; (2) implementation challenges and opportunities, including integration of the tool into clinical workflow; (3) adherence to and engagement with the tool, with suggestions for reminders and involvement of nurses; (4) perceived limitations and improvement of the screening tool, particularly the nutrition and sleep questionnaires; (5) feedback on the screening report and intervention emphasizing clarity and actionable guidance; and (6) perceived clinical value and opportunity costs. Insights from these discussions guided refinements of the digital tool.

**Conclusions:**

The findings support the tool’s relevance and inform its ongoing development. A feasibility study is planned prior to a randomized controlled trial.

## Introduction

In Canada, chronic preventable diseases cause 65% of all deaths [1], affect 44% of Canadians, and account for 58% of the annual health care budget [2]. Unhealthy lifestyle behaviors such as physical inactivity, excessive screen time, unhealthy diet, and poor sleep hygiene are key contributors to chronic preventable conditions, including obesity, diabetes, cardiovascular disease, several types of cancer, and psychological illnesses [3-9]. These behaviors often originate in childhood and tend to track into adulthood [9-11] and cluster [5,12-14].

Despite recommendations for systematic screening by several organizations, including the Canadian Task Force on Preventive Health Care [15-17], most children are not screened for lifestyle behaviors. Barriers include limited clinician time, competing priorities, fear of upsetting families, and low perceived self-efficacy of clinicians [18-22].

Digital preappointment screening can help overcome these barriers [23-26]. Compared to traditional methods, it is more time efficient, scalable, and generally well accepted by clinicians and families [23,24,26-32]. In pediatrics, digital screening, brief intervention, and referral to treatment (SBIRT) has helped address adolescent substance abuse [31,33-35] by identifying risky behaviors, initiating behavior change, and connecting patients to support resources [36,37]. Similar tools have been developed for parents to support childhood obesity in primary care [38].

Active stakeholder involvement is essential in developing screening and intervention tools [39-41]. Parents can help ensure the tool’s relevance and fit within real-life family contexts [42], whereas clinicians inform its feasibility and acceptability in practice [31,43,44]. For example, an intervention to support young adults with type 1 diabetes was codeveloped with patients, researchers, and health care professionals, helping identify priorities and concrete strategies to improve care [45].

This study sought to develop, alongside stakeholders, a tool to target health behaviors in children. Specifically, the primary objective of this study was to refine a screening and intervention tool using end user feedback obtained through focus groups [46]. This paper describes the codevelopment and refinement through structured focus groups with parents and clinicians of a self-administered digital preappointment screening and intervention tool targeting physical activity, sleep, nutrition, and screen time in children aged 2 to 12 years in general pediatric primary care.

## Methods

### Preliminary Data and Tool Development

This study is part of a larger research program that was initiated between October 2021 and November 2022, with a needs assessment conducted with 114 families at the Groupe de Médecine de Famille Universitaire Saint-Hubert, a suburban family medicine group [47]. This needs assessment confirmed clinicians’ interest in obtaining more information about children’s lifestyle behaviors, highlighted the prevalence of unhealthy behaviors in this population, and revealed parents’ interest in the development of a preappointment screening tool for lifestyle behaviors. The current manuscript describes the subsequent step of tool refinement through focus groups with parents and clinicians.

A first iteration of our SBIRT tool used three inputs: (1) literature on behavioral science frameworks, including nudges and comparisons that improve real-world decision-making [48,49] and health behavior change [49,50] supported by evidence on physical activity [51,52] and nutrition [53-55] in adults and promising pediatrics data [56]; (2) input from clinicians and family partners [25,35,57-59]; and (3) existing tools such as the RIPPLE (Resource Information Program for Parents on Lifestyle and Education) program, an eHealth SBIRT tool developed by Ball and van Mierlo [25,38,58,60] to prevent pediatric obesity.

More specifically, behavioral sciences were used to inform both the structure and the content of the tool given the promise of this domain to strengthen pediatric nutrition interventions. Insights into cognitive biases and parental motivation—which can be sought through the health behavior screening process by inquiring about motivation to change and beliefs about their children’s behaviors relative to group averages—can enable the development of more targeted and effective strategies that support the long-term adoption of healthy eating behaviors in children [49]. Although primarily focused on dietary habits, these strategies are broadly applicable to health behavior change. In this study, we sought to leverage these behavioral science insights through promising approaches such as personalized risk communication [61], the “fresh start effect” [62,63], implementation intentions [64,65], fostering internalization of healthy behaviors [66,67], and strengthening self-regulation [68-70]. The use of these frameworks seeks to enable enhanced parental engagement and facilitate the integration of behavior change into daily family life, notably by establishing a concrete and specific link between a health behavior and the action required to improve it, which may support the repetition of healthier behaviors and eventual automatization [49].

### SBIRT Content and Description

Following the standard approach in the literature [25,38,60], our tool consists of 3 main sections: screening questionnaire, brief intervention, and referral to resources (Table 1).

**Table 1:** Screening, brief intervention, and referral to treatment tool core components.

Components	Description
Screening	Parents complete a digital, self-administered questionnaire covering the family’s demographic information and the child’s health behaviors (physical activity, sleep, nutrition and screen time). The questions were informed by the results of a prior needs assessment.
Brief intervention	After screening, families receive a personalized report summarizing all behaviors and highlighting those not meeting national health guidelines. Feedback, codeveloped with Evolution Health (van Mierlo, CEO) and family partners, includes behavioral strategies (eg Specific, Measurable, Achievable, Relevant, Timebound (SMART) goals and nudges). Age-appropriate educational materials are emailed, and results are shared with clinicians to support counseling.
Referral to resources	For each behavior not meeting the guidelines, parents receive a curated list of community resources tailored to their child’s age and needs. These resources, selected with input from health care professionals and parent partners, are shared with parents based on the behaviors they indicate interest in changing on the brief, personalize report page.

### Tool Refinement: Focus Groups

The focus group discussions served the primary objective of the study: the development and refinement of our screening and intervention tool using end user feedback [46]. Specifically, the focus groups were intended to (1) gain insights into the needs of clinicians and parents regarding a digital tool aimed at promoting healthy behaviors; (2) explore participants’ past experiences with health technology; (3) integrate insights obtained from the first focus group into the tool development process; and (4) assess the perceived usability, relevance, acceptability, and accuracy of the developed tool. In the first and second focus groups, participants engaged with static mock-ups of the interface, which included the intended questions for each health behavior, and discussed and refined the visual outputs. This led to the fully operational screening and personalized intervention tool evaluated in the third focus group. During this focus group, participants interacted with the working interface, including the full screening questionnaire; immediate personalized feedback reports; comparative normative data displays; color-coded results across physical activity, sleep, nutrition, and screen time domains; and embedded links to actionable resources and follow-up guidance. The interactive tool is available on the operational DISCO platform [71].

### Participants and Recruitment Process

Two groups of participants were recruited from the Groupe de Médecine de Famille Universitaire (GMF-U) of Saint-Hubert: (1) primary care clinicians and (2) parents or legal guardians of children who are patients of the GMF-U of Saint-Hubert. Purposive and convenience sampling strategies were used to ensure rich, relevant data while promoting in-depth discussion.

Clinicians were recruited through an internal invitation sent by the management of the family medicine clinic. Parents were recruited via email invitations relayed by clinic staff to those with upcoming appointments for children aged 2 to 12 years. Eligibility criteria included being a parent or legal guardian of a child in that age range and being fluent in French or English.

### Data Collection

Three semistructured focus groups (August 2024, November 2024, and March 2025), each lasting 60 to 90 minutes, were conducted. Clinicians participated in person in August and virtually thereafter, whereas parents joined virtually in all sessions (via Microsoft Teams). Due to availability constraints, 8 sessions were held as individual interviews. One clinician and 1 parent participated in all 3 focus groups, whereas others joined a single session.

Discussions explored users’ experience with the digital health tool; perceived usability and relevance of the prototype; satisfaction; and suggestions for content, format, and delivery (Multimedia Appendix 1). Iterative refinement of the tool was based on feedback collected throughout the sessions. For example, after the first session, visual mock-ups were developed based on user expectations; the second session focused on reactions to various features (eg, feedback messages); and the third session examined acceptability and emotional responses to the tool. All data were collected and analyzed in French and translated into English for reporting.

Data collection continued until thematic saturation was reached, defined as the point when themes became repetitive across groups and no new themes emerged during iterative coding. At this stage, participants’ contributions reinforced and stabilized existing themes. In this study, saturation reflects internal coherence rather than statistical representativeness [72]. The same saturation criteria were applied to both parents and clinicians.

### Data Analysis

All focus group recordings were transcribed verbatim and reviewed for accuracy. An iterative, hybrid analysis—combining inductive and deductive approaches—was conducted. Deductive codes were elaborated using a published framework covering 7 constructs of usability, acceptability, and satisfaction (affective attitude, burden, perceived effectiveness, ethicality, coherence, opportunity costs, and self-efficacy) [73]. Furthermore, initial coding involved open coding aiming to identify emerging meaning units (ideas, experiences, and suggestions), followed by axial coding to identify conceptual relationships and central themes.

Data were analyzed at the group level using thematic analysis to describe and interpret patterns of meaning [74]. The process was managed using Microsoft Excel. To ensure coherence and transparency, the coder conducted systematic internal checks through repeated readings, comparison of the codes across cycles, and iterative refinement of the categories. Emerging themes were also discussed with the research team to strengthen analytic credibility [75]. Following a sequential cocreation approach inspired by the development of the RIPPLE pediatric obesity prevention tool [25,38,58,60], emerging themes from participants’ feedback guided refinements to the tool, including clarifying content, improving ergonomics, and simplifying usability.

### Ethical Considerations

This study received ethics approval from the research ethics boards of the Centre intégré de santé et de services sociaux de la Montérégie-Centre and the Sainte-Justine University Hospital Centre (application MP-04-2024-889 – DISCO – Focus Groups).

All participants provided informed consent prior to taking part and consented to the use and publication of their data. Each participant received a compensation of CAD $25 (US $18) per session.

Data were stored securely on the servers of Research Centre of the Sainte-Justine University Hospital Centre.

## Results

### Overview

Six discussions were conducted with a total of 8 participants, including 5 parents of children and 3 clinicians. Sessions were held either as individual interviews or dyadic discussions. All participants were female except for 1 clinician. Clinicians were family physicians with 5 to 6 years of professional experience. Participating parents had 1 to 3 children aged between 3 and 14 years.

### Potential of the Digital Tool for Promoting Healthy Habits in Pediatrics

#### Parental Experiences With Digital Health Tools

Participating parents reported previous use of various digital tools to support their children’s health, including platforms such as Dialogue (a telemedicine app) [76], smartwatches (for activity and sleep tracking), *Naître et grandir* (a government-sponsored parenting education website) [77], and *Rendez-vous santé Québec* (an online medical appointment–booking platform) [78]. Tools with simple, concrete guidance were particularly valued. One parent shared the following:

[The information is] written in a way that is very understandable for the general public, so it’s very simple, very concise, with concrete examples.

Several participants expressed concerns over tools perceived as overly commercial, simplistic, or lacking credibility. Some worried that digital tools disrupt the parent-child relationship:

I don’t necessarily want a tool, a robot, to replace that connection with my child.

They emphasized the importance of professional interfaces, quality assurance, and rigorous linguistic review in both French and English. One parent noted the following:

...small typos are fine, but blatant errors diminish the value of the questionnaire and makes it look less professional.... It makes the results seem less valid.

#### Identified Needs for a Digital Tool

Parents described specific content needs for a digital lifestyle intervention tool, notably the desire for tailored guidance on complex topics such as eating behaviors and sleep challenges. They emphasized the following:

If you don’t seek out information, you just do what you can, but you don’t really know what the best practices are [...] [guidance] should come directly to us, you know, so we don’t have to go looking for it.

#### Clinician Perspectives on Automation and Screening

Clinicians generally supported automated lifestyle habit screening to reduce clinical burden. One stated the following:

In a perfect world, it wouldn’t be the family doctor conducting lifestyle habit screening.

They saw value in the tool providing personalized information while underscoring the importance of follow-up dialogue:

Our role is then to discuss how to apply it concretely in daily life [...] like motivational interviewing.

Some participants supported broader automation, comparing the tool to mental health self-care platforms:

More programs designed to be accessible to the public, encouraging them to try self-care interventions. The more information you provide—especially if it’s increasingly personalized and from reliable sources—the better. I fully support that.

### Implementation Challenges and Opportunities

#### Workflow Integration

Clinicians highlighted several barriers to completing the tool in the clinic. Filling it out in the waiting room was seen as unrealistic due to time constraints, late arrivals, and the difficulty of managing young children. Additionally, receiving results via email limited clinicians’ ability to review them in time for the consultation.

To improve integration, clinicians strongly preferred embedding the tool in the electronic medical record, describing it as a straightforward and efficient solution. One noted the following:

...if it’s a result [in the electronic medical record], [...] we have no choice but to read it, otherwise, it will always stay highlighted as an incomplete task note. This definitely encourages clinicians to check it out.

#### Feasibility and Added Value in Clinical Practice of Use Prior to the Appointment

Participants agreed that it would be more practical to send the questionnaire before the appointment, ideally alongside the appointment confirmation, so that clinicians can review it beforehand and tailor the visit. Some emphasized the importance of doing so even for walk-in visits, where vulnerable families may be reached unexpectedly:

That’s often where we catch them, especially since parents usually come for regular check-ups up to the age of 3-4, and after that, we tend to lose them.

This suggests that the tool could help maintain contact with families beyond early childhood, when routine follow-ups become less frequent.

### Adherence to and Engagement With the Tool

#### Challenges

Maintaining long-term engagement, a prevalent issue in the digital health literature [79,80], was a shared concern. Both clinicians and parents noted that, after the age of 2 years, appointments become infrequent, making regular follow-up difficult. Motivation was seen as key: “I’d need to be fully committed,” said one parent. Engagement also tended to decrease with subsequent children:

...it’s nice when it’s your first child [...] then parents lose interest.

Clinicians saw value in a follow-up system to track engagement and identify families at risk of dropping off.

#### Suggested Solutions

##### Reminders Linked to Appointment

Implementing email notifications that encourage parents to schedule follow-up appointments to discuss lifestyle habits after long gaps between visits was suggested:

Email notifications could help.

##### Involvement of Nurse Practitioners

As they often see families more regularly, they could reinforce messages and ensure continuity.

##### Motivational Prompts and Progress Tracking

Parents welcomed the idea of periodic check-ins:

[If I am] asked, “Do you agree to receive the link again in a few weeks to check your progress?” Then, yes, I’d definitely do it.

##### Trusted Framing

Parents stressed the importance of the tool appearing credible and tied to a medical source (eg, physician’s office or public health institution).

##### Multichild Usability

A suggested addition was the ability to complete the tool for more than one child at a time.

### Perceived Limitations and Improvement of the Screening Tool

#### Sleep Habit Screening

Clinicians initially found the sleep section too limited, focusing mainly on duration with little attention to sleep hygiene. One noted that sleep problems often emerge indirectly, such as through academic or attention issues. A suggested addition was “Has the teacher reported any fatigue, sleepiness, or attention problems with your child?” to better capture underlying concerns.

#### Nutrition Screening

Both parents and clinicians found the initial emphasis on sugar-sweetened beverages (SSBs) too narrow. One parent noted the following:

There are many other aspects of nutrition that are more relevant to me than sugary drinks.

A clinician also observed that dietary discussions often go beyond SSBs, pointing out that “Parents are very concerned about the amount of food that is eaten” and that “it’s surprising how often families don’t eat together.”

Key nutrition-related challenges raised by parents included the following:

- Food exploration and parental education: exposure to disliked foods is still important, especially between the ages of 0 and 5 years
- Picky eating: a common source of stress in early childhood
- Time constraints and meal planning: balancing work and family limits time for healthy meals
- Teenager autonomy: teenagers make more independent and sometimes less healthy choices

#### Tool Revisions and Validation

After the first focus groups, the screening tool was revised to incorporate parent feedback. Sleep-related questions were revised to better capture sleep hygiene and overall sleep quality. While the original questionnaire included limited content, the updated version encompasses a broader range of dimensions, such as daytime sleepiness, napping patterns, regularity of bedtime routines and schedules, characteristics of the sleep environment (eg, independent sleep and presence of screens), sleep onset (autonomy and latency) and nighttime disturbances (eg, cosleeping behaviors). Similarly, the nutrition section was broadened to reflect overall dietary behaviors and the mealtime environment. Although it previously included a single item on SSB consumption, the revised version expanded to food-related behaviors and environment and, as such, included questions on meal regularity, family meal practices, dietary quality (eg, fruit, vegetable, and protein intake), beverage choices, screen use during meals, child involvement in food preparation, meal planning practices, and parental feeding strategies. Language was simplified, and examples were added for clarity (Multimedia Appendix 2).

In the second set of focus groups, these changes were well received. A clinician described the updated items as “excellent, very detailed,” noting the following:

Eating behavior is really interesting. [...] [and] relevant.

A parent highlighted the focus on “observable behaviors” and appreciated the educational aspect:

It tells me, “this is what a sleep routine is.” It kind of educates me at the same time as giving advice.

### Feedback on Screening Report and Intervention

#### Presentation and Content of the Screening Results

Participants responded positively to the screening report’s format and tone. Clinicians appreciated the inclusion of all behavior domains regardless of the results and the clear, color-coded layout:

Presenting all four sections, regardless of the results, is very positive [...] The positive approach to encouraging them and the available resources are very well designed.

The tool was considered simple and accessible for most families. The report’s emphasis on autonomy was also valued:

It’s really good to let the parent decide which area they want to be informed about because having 3 or 4 areas to work on [can be a lot], [...] [this way, they can] take small steps on their own with your recommendations.

#### Relevance and Usability of the Information

Parents appreciated receiving individualized and contextual data, especially charts comparing their children’s behavior with national guidelines and clinic averages:

The comparative aspect is still fun, because we say we shouldn’t compare ourselves, but at the same time, we all like to compare.

However, the phrase “Top 10% of the clinic’s most performing children” was criticized for being too competitive:

It should be about healthy lifestyle habits, not performance.

Opinions varied on the level of detail in the graphs: some wanted more specificity (eg, types of activities, frequency, and age group distinction), whereas others felt that the current indicators (individual scores, recommendations, and averages) were sufficient. The relevance and appropriateness of within-clinic comparisons were also questioned by 1 participant.

#### Intervention and Resources

Participants valued the clarity, concreteness, and relevance of the resources. One parent said the following:

It’s informative to know the impacts it has. The healthy goals. I also like seeing that, for example, the link for a tax credit provides information that we don’t have. The resources are clickable on one of the links. It’s very comprehensive, so it’s interesting.

They appreciated “immediate, applicable solutions,” such as links to recipes or planning tools. Suggestions included practical tips for limiting screen time and integrating local resources by postal code (eg, green spaces and cycling paths). The intervention was seen as supportive of gradual, realistic change:

Sometimes it’s nice to [...] take small steps by ourselves with the recommendations. I think that’s the whole point.

Overall, participants responded positively to the behavioral approaches included in the intervention.

#### Feedback on Encouragement Messages and Follow-Up

Tailored follow-up messages were seen as essential, particularly for families whose results did not improve. One parent noted the following:

...if [it has not worked] what can we do to help these families if it hasn’t worked? [...] maybe we haven’t seen improvements because you’ve maintained the same behavior. Did you know that there is another approach or reference?

Personalized resources, whether to maintain progress or adjust strategies, were deemed essential to sustaining motivation.

### Perceived Clinical Value and Opportunity Costs

Clinicians found the refined version of the tool efficient and user-friendly:

I really like that the recommendations for clinicians take only 20 seconds to read.

Its brevity was seen as a strength that facilitated dialogue:

It opens the door for discussion.

Both clinicians and parents viewed it as time-saving and accessible. One parent noted the following:

It’s straightforward. [...] you don’t really feel like filling out long tools while waiting in the clinic. I think you mentioned 15 minutes to complete it, but it felt more like 5.

Clinicians highlighted three key strengths of the tool: (1) time savings (“It will save us time...and it aligns with a motivational interviewing approach since you’ve already started the work”), (2) initiation of the behavior change process (“prompting them to come with an answer, a rationale, or a potential solution at the next appointment. I think that could be beneficial”), and (3) increased parental engagement (“I think it’s good to send this to all parents in any consultation context; I think it can be a good opportunity to just raise their awareness”).

However, the tool’s perceived value varied by family. Parents with prior knowledge or motivation felt that it might be too basic, whereas those with less awareness could find it overwhelming:

If it’s for families who are already looking to improve behaviors, it might not go far enough for them. But for a family that really has no idea and needs to make changes, it might feel overwhelming.

## Discussion

### Principal Findings

Through our focus groups aimed at refining our digital screening and intervention tool to promote healthy lifestyle habits in children, we found that the tool’s key features, such as personalized, immediate, and normative feedback and access to concrete resources, were consistently valued by participants and align with known drivers of engagement and sustained behavior change [81,82]. Overall, parents reported feeling that the tool’s questions were relevant and that the approach to offering support in behavior change and tailoring follow-ups, as well as the resources offered, was helpful and acceptable. Clinicians found the refined version of the tool efficient and user-friendly, and while they expressed concerns about barriers to completing the screening in the clinic, they felt that it was relevant to use a tool of this nature to perform lifestyle behavior screening between appointments and that the data presented were insightful, especially as clinical follow-up becomes less frequent as children grow older.

The elements of the intervention that were guided by behavioral science principles, such as comparisons to peers and norms and personalized feedback, were well accepted by participants. The persuasive potential of behavioral sciences relies on modifying choice architecture (ie, the decision-making context in which individuals act [83]). These interventions aim to guide behaviors toward outcomes considered beneficial while preserving freedom of choice as no options are removed or restricted [84]; therefore, guiding behaviors without unduly influencing or exerting coercive pressure that may limit informed decision-making [85]. In our development of the tool and implementation of strategies from behavioral science frameworks, we carefully considered the balance between behavioral effectiveness and respect for autonomy [86] and, through our focus group discussions, ensured that we elicited any autonomy-related concerns from stakeholders about the intervention; none were raised.

Overall, our study highlights the potential of a codeveloped digital tool for screening and promotion of healthy lifestyle habits in pediatric primary care. The participatory approach enabled iterative refinement based on clinicians’ and parents’ input, ensuring high acceptability, usability, and relevance (Multimedia Appendices 2 and 3).

While the integration of stakeholders supports participatory design, organizational factors such as timing of distribution, follow-up mechanisms, appointment coordination, and communication between families and professionals must be addressed for effective clinical implementation. These align with known implementation challenges in feedback-based interventions, where mode, frequency, and source of feedback influence the impact [87]. Future phases of this research project will leverage the early insights gleaned in this study and seek to address them to elucidate the most effective path forward.

As mentioned by participants, equitable access remains challenging, especially for families facing structural barriers (eg, financial constraints, fragmented services, and logistical difficulties). To address this, the tool was codeveloped with parents and clinicians, and tailored strategies with concrete support were prioritized to reduce contextual barriers and foster engagement [88]. Still, the recruitment process may have introduced selection bias as participating clinicians were likely more engaged in lifestyle-related screening and some parents may have been more motivated or available. The small sample size of this study and the self-selected nature of participants (all from a single clinical site) further limit the generalizability of the tool in development.

This could limit the tool’s applicability to less engaged or more vulnerable populations—the very groups for whom equitable access is most critical. However, the primary objective was to gain insights into key concepts essential for tool development rather than produce exhaustive results. Future phases of this program of research will involve implementing and evaluating the tool in more generalizable settings, including through open-label testing in larger and more diverse populations, and using the screening and brief intervention tool to facilitate access to lifestyle behavior screening, all while collecting data on feasibility and acceptability from a more diverse population, leading to eventual implementation in clinical and other pediatric settings.

Verbatim translation from French into English may have introduced bias; efforts were made to preserve meaning within feasibility constraints [89]. Additionally, the sample size and the mix of focus groups and individual interviews may have influenced interaction dynamics, although the consistency of perspectives across formats strengthens the credibility of the findings [90].

Despite these limitations, this study lays a strong foundation for future work. Subsequent phases will include a feasibility study and broader engagement with stakeholders to assess the tool’s adaptability. This will be followed by a pilot study and a randomized controlled trial to rigorously evaluate the tool’s effectiveness in improving pediatric health outcomes.

### Conclusions

To our knowledge, no similar preappointment screening tool exists in general pediatric care. This study provides early evidence on the feasibility and relevance of a codeveloped digital tool to promote healthy lifestyle behaviors in pediatric primary care. Its participatory design and user-valued features support its potential for future evaluation and implementation. This work represents the initial phase of its development. Future phases of the project will evaluate tool completion rates, repeat engagement, uptake across clinic-specific instances, and workflow integration metrics. Secondary outcomes will include changes in parent-reported child lifestyle behaviors across physical activity, sleep, nutrition, and screen time domains. These next-stage outcomes are guided by the broader implementation principle that, while there are an increasing number of validated tools and evidence, dissemination remains a major barrier to real-world reach and impact. In this context, infrastructure determines equity and access, and future phases will specifically assess the scalability of deployment across various settings, including pediatric clinics, family medicine groups, and nonclinical environments such as schools and community centers, particularly to reach children and families who may not otherwise have timely access to traditional care settings [91].

## Appendix

Multimedia Appendix 1 Detailed guides of the different focus group discussions.

Multimedia Appendix 2 Detailed version of the screening questionnaire included in the DISCO tool.

Multimedia Appendix 3 Screenshots of interfaces and visuals included in the DISCO tool.

## Abbreviations

GMF-U: Groupe de Médecine de Famille Universitaire
RIPPLE: Resource Information Program for Parents on Lifestyle and Education
SBIRT: screening, brief intervention, and referral to treatment
SSB: sugar-sweetened beverage

## Data Availability

The datasets generated or analyzed during this study are not publicly available due to confidentiality concerns given the small number of focus group participants but are available from the corresponding author on reasonable request.
