# Supplementary material for: Codevelopment of a Digital Screening and Intervention Tool to Improve Lifestyle Habits in Children: Focus Group Study With Parents and Clinicians
Source: JMIR Pediatr Parent. 2026 Jun 26;9:e84304. doi: 10.2196/84304 (PMC13354948; doi:10.2196/84304)
Supplement: Multimedia Appendix 1 [file pediatrics_v9i1e84304_app1.docx]

**1. Clinicians – Focus Group 1**

First, did you participate in the first phase of this project, between 2021 and 2023, during which we provided you with a brief report on the lifestyle habits of clinic patients who completed the screening questionnaire?

- **If no:**
  - How do you think this tool might affect your practice as a clinician?
  - Would you consider integrating it into your work?
    - If yes, what impact do you think it would have on your workflow?
    - Do you think it would give you more opportunities to discuss children’s lifestyle habits with families during clinic visits than before?
- **If yes:**
  - Did you use the lifestyle reports?
  - Can you briefly explain how you integrated them into your work?
    - If yes, what impact did it have on your workflow?
    - If not, can you explain why you did not use it? Do you have suggestions for improving the screening process and its integration into your clinical work?
  - What kind of questions did families ask after completing the screening questionnaire?
  - Did you face refusals or negative comments from families when initiating a conversation about the screening results?
    - If yes, please describe these situations and the negative comments expressed.
  - Did families make suggestions regarding the project?
    - If yes, please describe the suggestions received from families about the project.
  - After completing the screening questionnaire, would you say that you discussed children’s lifestyle habits with families during clinic visits more than before, less than before, or that it did not change your practice in this regard?
- In the preliminary data, all participants reported that their child had adequate sleep habits. What is your perception of sleep quality in your patients aged 2 to 12?
  - Do you think it is relevant to discuss sleep hygiene with parents using this tool?
- How much time do you spend discussing healthy lifestyle habits with families?
- Regarding healthy lifestyle habits, how do you see the division of tasks between a tool like this one and yourself for screening and intervention?
  - To what extent could automation of screening and intervention through the tool be beneficial in your daily practice?
  - What level of automation of screening and intervention would you consider acceptable and beneficial?
- Do you have suggestions to make this tool more effective in promoting healthy lifestyle habits?
- Do you have suggestions for improving the process of this project and its integration into your clinical work?
- Based on your responses in this focus group, along with information provided by parents and the questionnaire, we will develop a prototype of the intervention tool to present to you in the next focus groups. Do you have any questions or suggestions regarding the project process and the development of this tool?

**2. Parents – Focus Group 1**

*Experience using technology for a health-related topic:*

- Have you ever used technology (phone, tablet, computer…) for a health-related topic, for example, having telemedicine appointments or using a smartwatch to track your health?
  - Can you give me an example of how you have used technological tools to support or manage your child’s health?
- Can you tell me about technologies you found useful to support behavior change (for example, to better understand or change eating, smoking, or drinking habits)?
- **[For non-users].** Were you aware of such technology or apps that help with behavior change? Are you interested in this type of tool?
  - Can you tell me why or why not?
- What elements, resources, or features offered by these technologies motivate you to use them?
  - On the contrary, what elements, resources, or features offered by these technologies motivate you to stop using them?

*Questions about using technology to modify the child’s behavior:*

- Have you ever used this type of technology with your child/for their lifestyle habits?
- What concerns would you have about using a mobile health technology like this to help your child manage their daily routine?

*Experience with the DISCO screening questionnaire:*

- First, did you participate in the first phase of this project, between 2021 and 2023, during which we sent you a screening questionnaire before your appointment with your clinician? (The screening questionnaire used in the first phase will be shown to participants.)
- **If yes:**
  - How did you feel when responding to this questionnaire?
  - Were there any questions you did not want to answer?
  - Do you have suggestions for improving the screening process and its integration into the clinic?
- **If no:**
  - Are there any questions you would not answer? If so, which ones and why?
  - Can you tell me what resources you would need or could use to better help you promote healthy habits for your child?
- How would you feel as parents about these types of questions?
  - In the past month, how many times per day/week/month has your child drunk a beverage other than milk or water?
  - On a scale of 1 to 5, how ready are you to increase your child’s level of physical activity?
  - On a scale of 1 to 5, how good of a time is it for you to increase your child’s level of physical activity?
- Currently, the questionnaire addresses four lifestyle habits (sleep, diet, screen time, and physical activity). Would you like to see other lifestyle habits included in the tool? If yes, which ones and why?
- Based on your responses in this focus group, along with information provided by clinicians and the questionnaire, we will develop a prototype of the intervention tool to present to you in the next focus groups. Do you have any questions or suggestions regarding the project process and the development of this tool?

**3. Clinicians – Focus Group 2**

- In the first focus group, we discussed the best ways to identify children’s sleep habits. We therefore created new questions that may better reflect these habits. I will read each of the sleep-related questions to you. Please indicate for each question whether it seems relevant to you, and if you have suggestions or comments. Do you think these questions would be more relevant and useful for your work?
- (We will show them the sleep screening questionnaire.)
  - Do you think it would be useful for this tool to serve as a point of contact with families to assess their needs between scheduled appointments?
    - If yes, how would you envision using it?
    - If not, would you still use the information on families’ health behaviors between appointments?
    - Do you think it would be relevant and feasible to use this point of contact to encourage parents to book an appointment for a lifestyle habits assessment?
- What do you think about how the information is presented?
- Do you have suggestions for the format or presentation of lifestyle information?
  - Are there any types of information you would have liked to see displayed?
- Do you think this type of tool would be useful in your practice?
  - Does this tool meet your needs as a clinician?
  - Which aspects of the tool are most useful for you in your practice?
- Do you think using this tool will make your appointments shorter or longer?
  - If longer, why?
  - If shorter, why?
  - How would this change what you discuss with families during appointments?
- Overall, do you think the tool seems easy to use?
  - If not, what makes it complicated?
- Are there elements you would like to see added or improved in the tool to better meet your needs?
  - What should this tool do to support you in your work?
- Do you think you would use patient reports generated by the tool?
  - If yes, how often would you consult them?
    - Can you describe how you would use these reports in your practice?
  - If not, why not?
    - If not, what alternative would you prefer?
- Would you feel comfortable using the tool’s results in appointments with patients?
- If lifestyle results could be integrated into the patient’s medical record, would you find that relevant?
  - If yes, what information would you want added to the record?
  - If not, how would you prefer to receive the information?
  - Do you use an electronic medical record?
- Do you regularly consult patients’ medical records before or between appointments?
  - If not, would it be possible for you to systematically review these records to track patients’ lifestyle progress?
  - At what point would you use the information provided by this tool?
- Based on the information collected in our focus group and our discussions with parents, we will adjust the prototype of the intervention tool we presented today to better meet your needs and expectations. We will present you with the updated version in the next focus group. Do you have any questions or suggestions regarding the project process and the development of this tool?

**4. Parents – Focus Group 2**

*Follow-up from first focus groups:*
We want to inform you that your needs for additional resources to manage your emotions, your child’s emotions, or to support academic performance have been taken into account after the first focus group. These resources will be included in the tool.

During the first focus groups, several participants expressed that the questions on sugary drink consumption may not adequately fit or meet your needs regarding your child’s nutrition.

- Can you tell us which aspects of nutrition represent challenges in your daily life as parents?
- Are there aspects of nutrition for which you would like more support or resources?
- Nutrition includes two dimensions: the quantity and quality of foods consumed, as well as the eating environment and eating behaviors. Do you face difficulties with this second dimension regarding your child’s eating?
- I will now mention different topics related to children’s nutrition, and I will ask you to tell me whether you would like to know more, talk about it with your clinician, receive support on this topic, or if it is not relevant for you and your child.
  - Frequency of snack consumption
  - Restricted food preferences
  - Mealtime environment (eating with family vs. alone, in front of the TV, always eating at the same time, etc.)

*Presentation of visuals:*

- What do you think about how the information is presented?
  - How does it make you feel?
- Which aspects of the tool would be most useful to you in promoting your child’s healthy lifestyle habits?
- Do you feel the tool meets your specific needs as parents?
  - Why?
  - Why not?
  - If not, what are your needs related to promoting healthy habits in your child?
- Are there specific features of the tool you find particularly useful or not useful?
  - Are there any aspects you find stigmatizing?
- Are there elements you would like to see added or improved in the tool to better meet your needs?
  - Are there types of resources or advice you would have liked to see displayed?
- Do you find the information provided by the tool clear and organized?
  - Did you find the tool’s design aesthetically appealing?
- When asked to evaluate this tool, what elements are most important in judging its quality? Could you name criteria or qualities that influence your evaluation?
  - How does this tool meet those criteria or qualities?
- Overall, do you think the tool seems intuitive?
  - If not, what makes it difficult to understand?
  - Do you have comments regarding the platform’s clarity or ease of use?
- Based on the information collected in our focus group and our discussions with clinicians, we will adjust the prototype of the intervention tool we presented today to better meet your needs and expectations. We will present you with the updated version in the next focus group. Do you have any questions or suggestions regarding the project process and the development of this tool?

**5. Clinicians – Focus Group 3**

- Following our last focus group, we modified the tool to meet your needs and expectations.
  - Do you notice any changes in the tool?
  - Do you notice any elements of the tool you previously appreciated that have been removed?
- Do you plan to incorporate this tool into your clinical practice?
  - Do you have concerns about integrating this tool into your clinical practice?
  - Do you think you will enjoy using this tool with patients?
  - Do you think this tool is the right approach to improving lifestyle habits?
- If not, what would you suggest?
- Do you think families will easily adopt this tool?
  - How do you think parents will react to this tool?
- Are there specific features of the tool you find particularly useless?
  - Are there aspects of the information that you think could be perceived as stigmatizing for patients?
- Imagine we send this feedback to parents. How do you think they would feel receiving it? (We will show them examples of encouragement messages or screening questionnaire feedback reports.)
  - Would you add any other information?
- Do you find the information provided by the tool relevant and helpful?
  - Do you think the digital tool adds value for the time and effort you invest in using it?
  - Are there aspects of the information that could be improved or developed?
- In your opinion, are there improvements that could ensure the acceptability and effectiveness of this tool?
  - What modifications, additions, or removals would encourage your regular use of this tool?
  - Are there additional features you would like to see added?
  - Do you have any comments, suggestions, or questions to share?

**6. Parents – Focus Group 3**

- Following our last focus group, we modified the tool to meet your needs and expectations.
  - Do you notice any changes in the tool?
  - Do you notice any elements of the tool you previously appreciated that have been removed?
- Can you describe your overall perception of this tool?
  - Does the screening and intervention tool meet your needs and expectations?
  - Are there specific features of the tool you find positive or negative?
- Which aspects of the tool do you find most acceptable?
  - Are there aspects of the tool you find unacceptable or unsatisfactory?
- Imagine we send this feedback to parents. How do you think they would feel receiving it? (We will show them examples of encouragement messages or screening questionnaire feedback reports.)
  - Would you add any other information?
- Do you find the information provided by the tool relevant and helpful?
  - Are there aspects that could be improved or developed?
- Do you think the digital tool adds value for the time and effort you invest in using it?
- In your opinion, are there improvements that could ensure the acceptability and effectiveness of this tool?
  - What modifications, additions, or removals would encourage your regular use of this tool?
  - Are there additional features you would like to see added?
- Do you have any comments, suggestions, or questions to share?
