# Supplementary material for: Codevelopment of a Digital Screening and Intervention Tool to Improve Lifestyle Habits in Children: Focus Group Study With Parents and Clinicians
Source: JMIR Pediatr Parent. 2026 Jun 26;9:e84304. doi: 10.2196/84304 (PMC13354948; doi:10.2196/84304)
Supplement: Multimedia Appendix 2 [file pediatrics_v9i1e84304_app2.docx]

**Welcome to DISCO!**
**Study: Development of a rapid screening and intervention tool to improve children’s lifestyle habits.**

Welcome to the webpage of the questionnaire about the development of a rapid screening and intervention tool to identify and promote healthy lifestyle habits in children. As a parent or legal guardian of a child aged 2 to 12 years, you are invited to take part in this study on children’s lifestyle habits.

**Objective:**
The goal of this study is to develop a rapid screening and intervention tool that can better identify lifestyle habits (physical activity, nutrition, screen time, and sleep) in children aged 2 to 12 years. This tool will help provide a better understanding of children’s lifestyle habits so that physicians and other health professionals can better promote healthy lifestyle behaviors.

**Participation:**
It will take about 15 minutes to complete the questionnaire. Please answer the questions to the best of your knowledge based on your experience and perceptions. There are no right or wrong answers.

**Benefits:**
You will not receive any immediate direct benefit from participating in this study. However, by completing the questionnaire, you will provide essential information to your child’s physician and contribute to improving healthcare professionals’ ability to encourage healthy lifestyle habits. If you indicate that you are interested in receiving information or discussing these lifestyle habits with your child’s physician, you will receive support in adopting healthy lifestyle habits, which could have a positive impact on your child’s health.

**Risks and Inconveniences:**
The main risks are the time required to complete the questionnaire and possible discomfort in sharing information about your child’s health behaviors. Data from this study will be collected and transmitted electronically. As with all electronic data, there is a risk of data loss or theft.

**Confidentiality:**
Participation in this study is voluntary and your responses are anonymous and confidential. The results of the questionnaire will be shared only with your child’s physician and the research team, and will be added to your child’s medical record. The research team will have access to the collected data, which will be de-identified so that your responses cannot be linked back to you or your child. The researchers are part of Dr. Olivier Drouin’s team at the Sainte-Justine University Hospital. All measures will be taken (data de-identification, collection and storage on a private password-protected server) to ensure the confidentiality of participant information. Responses to the questionnaire will be kept for five years after the end of the project, after which they will be destroyed.

**Consent:**
By completing this questionnaire, you consent to participate in this study. You are completely free to refuse to participate and may decide at any time to stop answering the questions. If you decide not to participate, this will not have any negative consequence for you or your child’s medical care.

The Research Ethics Board of the CISSS de la Montérégie-Centre has approved this project and will oversee it. We are also committed to submitting for approval any revisions or modifications to the research protocol or the consent form. If you wish to contact this committee, you may do so by calling the coordinator at 450-466-5000, extension 3894.

**Contacts:**
For any questions, you may contact:

- **Principal Investigator:** Dr. Olivier Drouin
  - Phone: (514) 345-4931, extension 4226
  - Email: olivier.drouin.hsj@ssss.gouv.qc.ca
- **Commissioner for complaints and service quality, CISSS de la Montérégie-Centre**
  - Phone: (450) 466-5434

**Acknowledgements:**
Thank you for your participation and valuable time. Your responses are important.

**Do you consent to participate in this study?**
[Yes] [No]

**General Information**

The following questions will help us verify your eligibility for this study and ensure the information is shared correctly with your child’s clinician.

- D1 What is your child’s date of birth?
- D2 What are your child’s initials?
- D3 What is your relationship with the child?
- D4 What is your email address?

**Physical Activity (ages 2 to 5)**

The following questions concern physical activity, defined as any activity that increases heart rate and causes shortness of breath. This includes sports, school activities, playing with friends, or walking to school.
Examples: running, brisk walking, dancing, swimming, roller skating, cycling, playing soccer, basketball, or football.

We are aware that external factors (e.g., school, time of year) may influence your responses. Please answer as precisely as possible in this section and the following ones.

**Sports and structured physical activities**

- PA1 On a typical weekday (Monday–Friday), how much time does your child spend on organized physical activities or sports (e.g., swimming, soccer, gymnastics)?
  Minutes/day: ___
- PA2 On a typical weekend day (Saturday and Sunday), how much time does your child spend on organized physical activities or sports?
  Minutes/day: ___

**Unstructured/free physical activity**
During a typical weekday (Monday–Friday), how much time does your child spend being active or playing (e.g., in a gym, during recess, in unstructured free play, in a park, going for a walk), excluding organized activities?

- PA3 During daycare/school and after-school care (minutes/day): ___
- PA4 Outside daycare/school and after-school care (minutes/day): ___

**Sleep (ages 2 to 4)**

- S1 Is your child sleepy during the day? [Always, Often, Sometimes, Rarely, Never]
- S2 Does your child take a nap? [Never, Rarely, Sometimes, Often, Always]
- S3 Do you have a bedtime routine (e.g., brushing teeth, putting on pajamas, dimming lights, reading a story)? [Never, Rarely, Sometimes, Often, Always]
- S4 Is your child’s bedtime the same every night? [Never, Rarely, Sometimes, Often, Always]
- S5 Does your child sleep in their own room? [Never, Rarely, Sometimes, Often, Always]
- S6 Does your child fall asleep alone in their room? [Never, Rarely, Sometimes, Often, Always]
- S7 On average, how long does it take your child to fall asleep? ___
- S8 Does your child join you in your bed during the night? [Always, Often, Sometimes, Rarely, Never]
- S9 Is there a screen (tablet, phone, television, etc.) in your child’s room? [Always, Often, Sometimes, Rarely, Never]

**Nutrition**

- B1 Are meals eaten at regular times? [Never, Rarely, Sometimes, Often, Always]
- B2 Are meals at home eaten as a family? [Never, Rarely, Sometimes, Often, Always]
- B3 How often does your child’s plate contain fruits and vegetables? [Never, Rarely, Sometimes, Often, Always]
- B4 Does your child’s plate contain protein-rich foods (meat, chicken, fish, eggs, yogurt, cheese, legumes, tofu)? [Never, Rarely, Sometimes, Often, Always]
- B5 Is water the main beverage during meals? [Never, Rarely, Sometimes, Often, Always]
- B6 Are all screens turned off during meals? [Never, Rarely, Sometimes, Often, Always]
- B7 Does your child participate in choosing and preparing foods? [Never, Rarely, Sometimes, Often, Always]
- B8 Do you plan meals for the week in advance? [Never, Rarely, Sometimes, Often, Always]
- B9 Do you use food as a reward or punishment for your child? [Always, Often, Sometimes, Rarely, Never]
- B10 Is your child a picky eater or small eater? [Always, Often, Sometimes, Rarely, Never]
- B11 Does your child often complain of being hungry between meals? [Always, Often, Sometimes, Rarely, Never]

**Screen Time and Sedentary Behaviors**

The following section is about leisure screen time in your child’s free time. Please do not include screen time related to school or homework.

This includes television, smartphones, computers, video games (Playstation, Xbox, Nintendo DS, Switch), tablets (iPad), as well as time spent watching movies, texting, emailing, or browsing social media.

In general, how much time does your child spend per day on screens? Please provide the total cumulative time spent using screens for all leisure activities during a typical day (in minutes).

- ST1 Weekdays: minutes/day ___
- ST2 Weekends: minutes/day ___

How many days per week does your child use screens within 30 minutes:

- ST3 Immediately after waking up in the morning? [0–7 days]
- ST4 Immediately before going to bed at night? [0–7 days]

**Next Steps**

Here are some areas you might consider working on (check the box for the behavior you would like to prioritize changing):
[List of behaviors not meeting national recommendations]
