# Supplementary material for: Codevelopment of a Digital Screening and Intervention Tool to Improve Lifestyle Habits in Children: Focus Group Study With Parents and Clinicians
Source: JMIR Pediatr Parent. 2026 Jun 26;9:e84304. doi: 10.2196/84304 (PMC13354948; doi:10.2196/84304)

**1. Summary of personalized results based on the responses provided**
The results related to each behavior are presented to families; here, only the charts concerning physical activity and nutrition are shown.


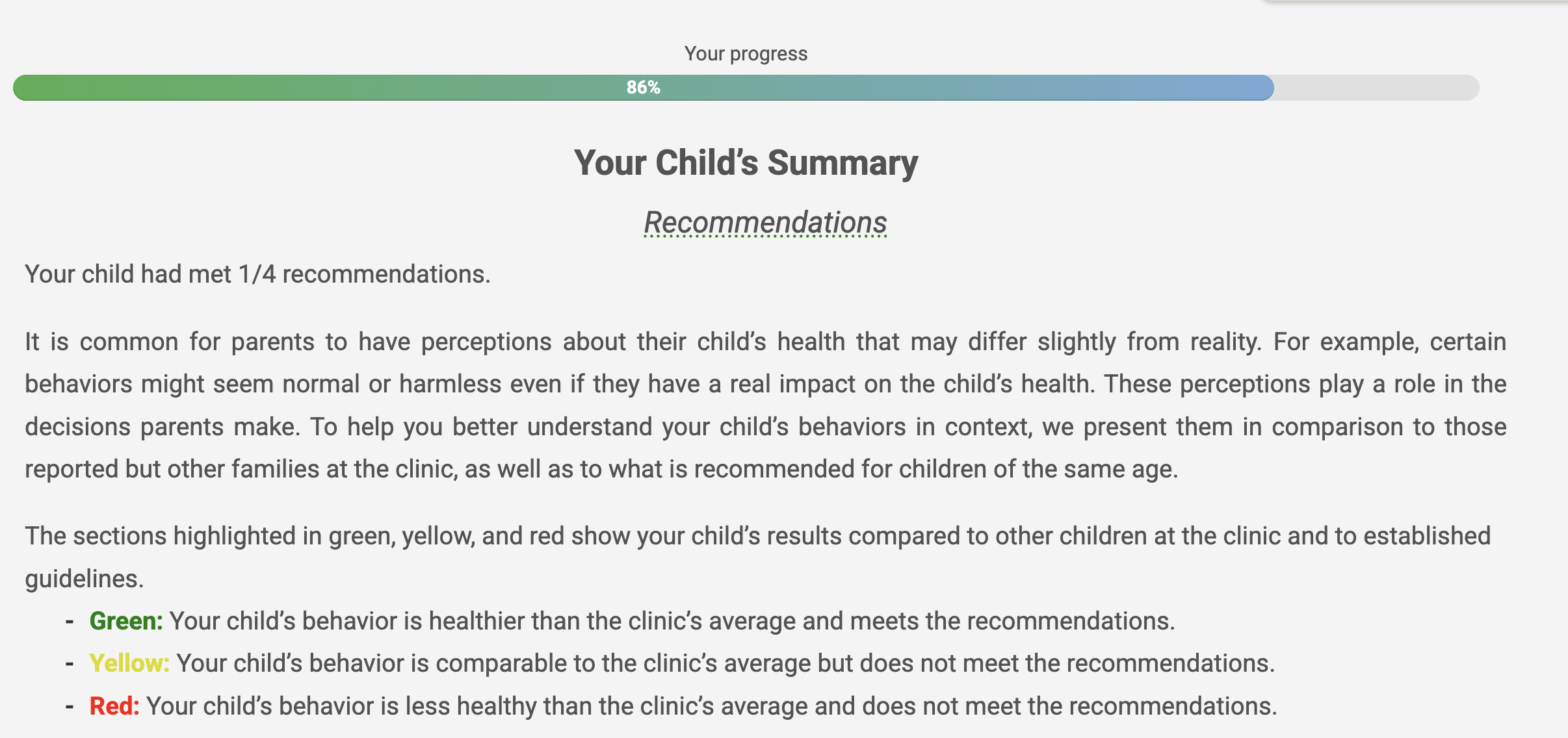


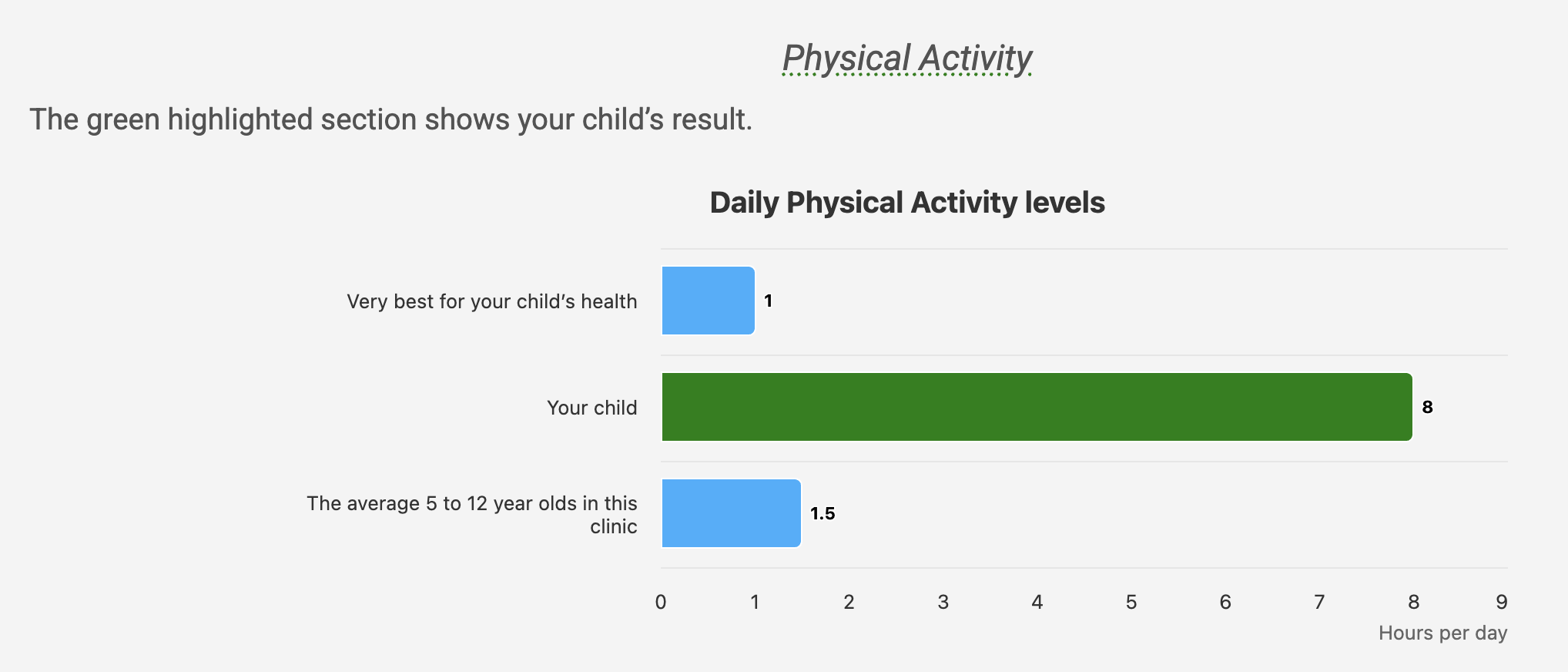


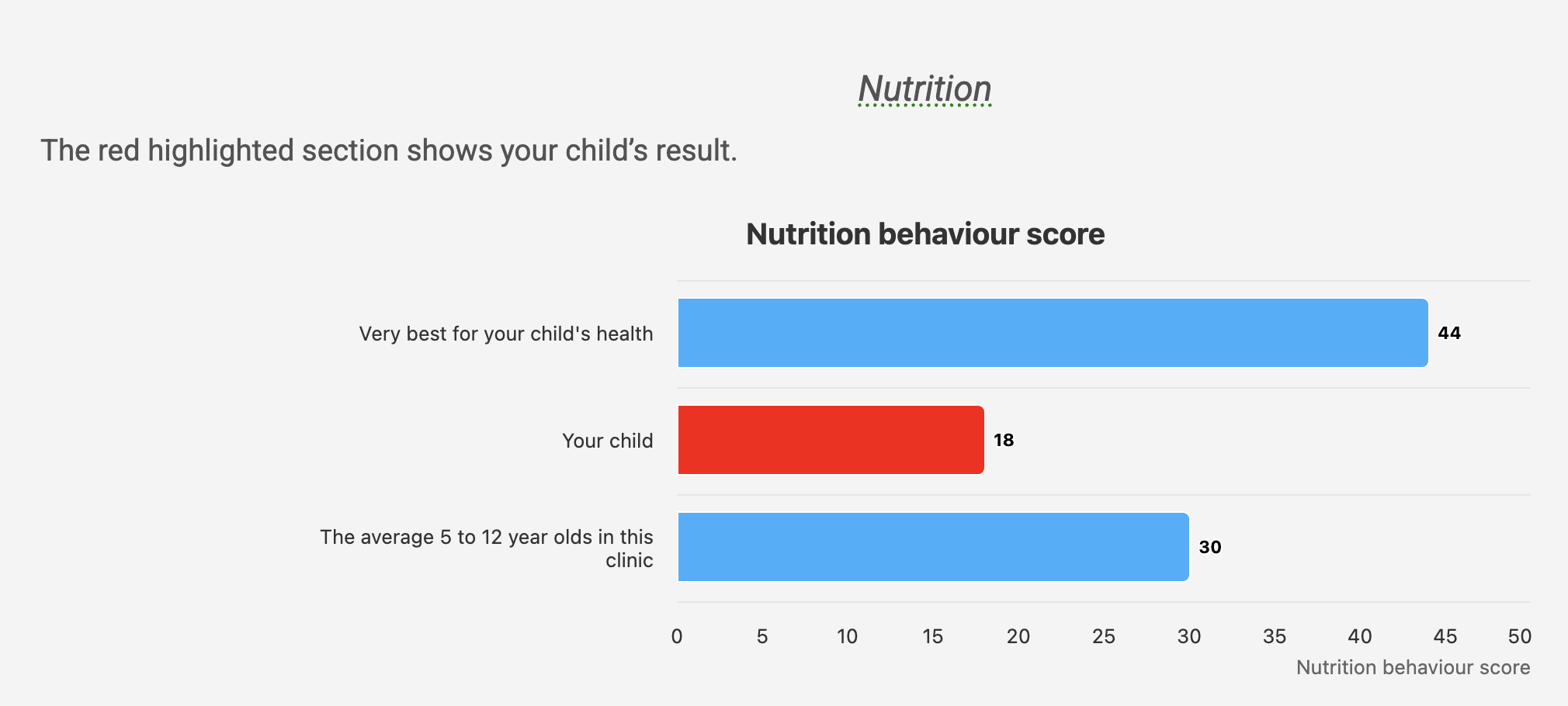


**2. Example of an intervention proposed to improve nutrition**


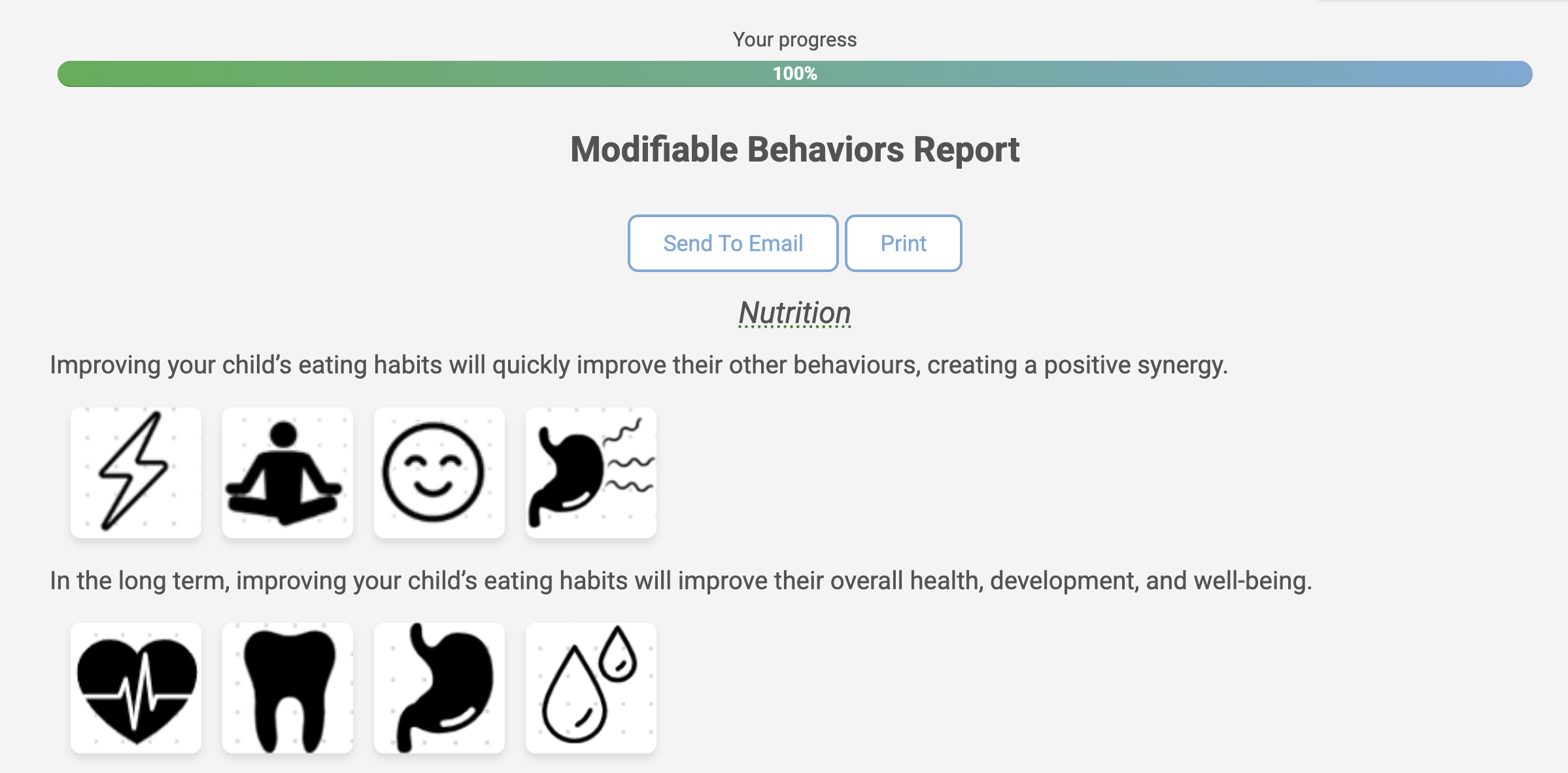


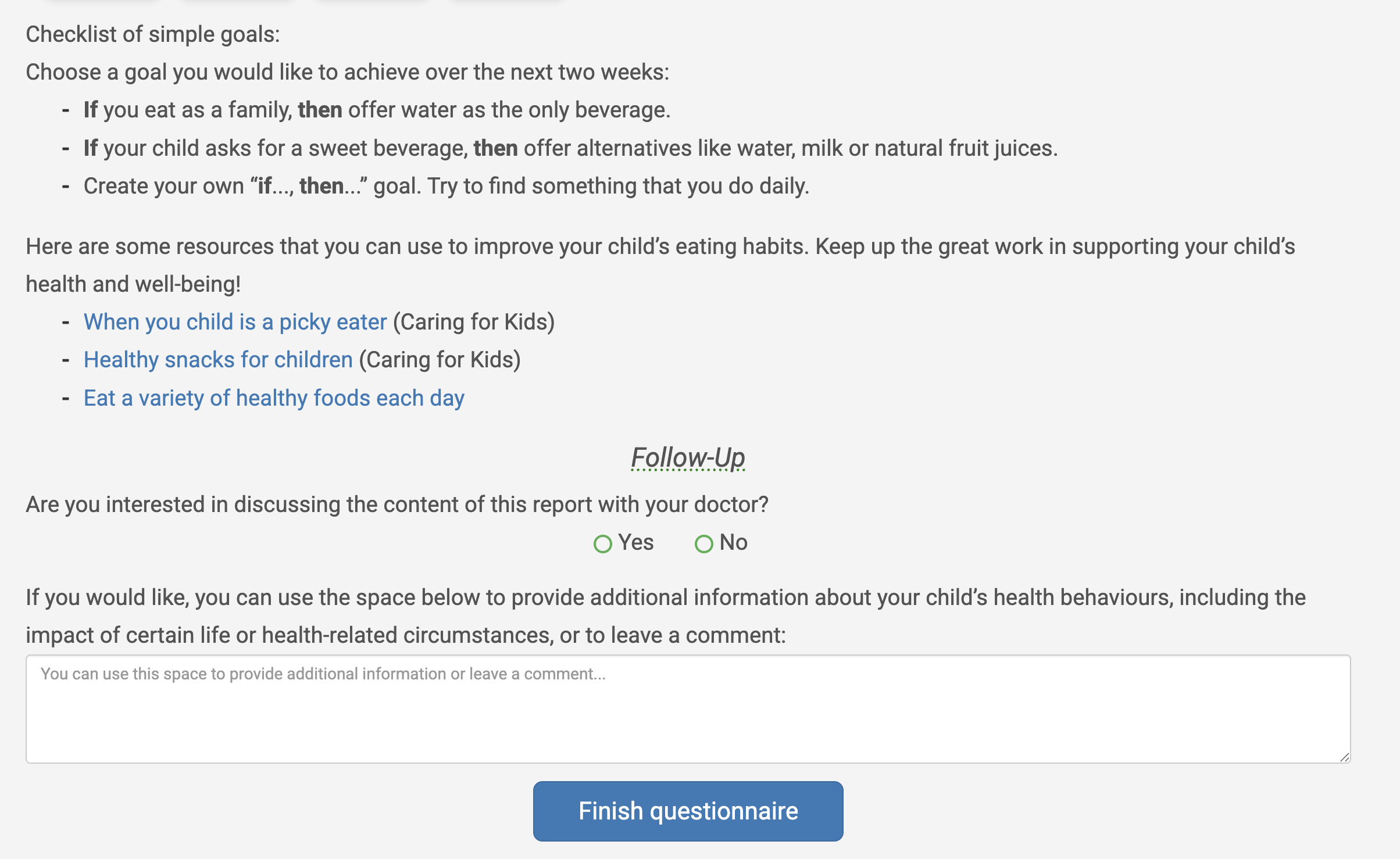

Supplement: Multimedia Appendix 3 [file pediatrics_v9i1e84304_app3.docx]
